# Determinants of Longitudinal Changes in Cardiometabolic Risk in Adolescents with Overweight/Obesity: The EVASYON Study

**DOI:** 10.3390/nu14153241

**Published:** 2022-08-08

**Authors:** Miguel Martín-Matillas, Dinalrilan Rocha-Silva, Abel Plaza-Florido, Manuel Delgado-Fernández, Amelia Marti, Pilar De Miguel-Etayo, Luis A. Moreno, Ascensión Marcos, Cristina Campoy

**Affiliations:** 1 PROFITH “PROmoting FITness and Health through Physical Activity” Research Group, Sport and Health University Research Institute (iMUDS), Department of Physical Education and Sports, Faculty of Sport Sciences, University of Granada, 18071 Granada, Spain; 2 Department of Physical Education and Sports, Faculty of Sport Sciences, University of Granada, 18071 Granada, Spain; 3 PA-HELP “Physical Activity for Health Promotion, CTS-1018” Research Group, Sport and Health University Research Institute (iMUDS), Department of Physical Education and Sports, Faculty of Sport Sciences, University of Granada, 18071 Granada, Spain; 4 Department of Nutrition, Food Sciences and Physiology, University of Navarra, 31008 Pamplona, Spain; 5 Navarra Institute for Health Research (IdiSNA), 31008 Pamplona, Spain; 6 Physiopathology of Obesity and Nutrition Networking Biomedical Research Centre (CIBERobn CB12/03/30002), Spanish National Institute of Health Carlos III, 28029 Madrid, Spain; 7 Physiopathology of Obesity and Nutrition Networking Biomedical Research Centre (CIBERobn CB15/00043), Spanish National Institute of Health Carlos III, 28029 Madrid, Spain; 8 Growth, Exercise, Nutrition and Development (GENUD) Research Group, Faculty de Ciencias de la Salud, University de Zaragoza, Institute Agroalimentario de Aragón (IA2) and Institute de Investigación Sanitaria de Aragón (IIS Aragón), 50009 Zaragoza, Spain; 9 Department of Metabolism and Nutrition, Institute of Food Science, Technology and Nutrition (ICTAN), Spanish National Research Council (CSIC), 28040 Madrid, Spain; 10 Department of Pediatrics, School of Medicine, University of Granada, 18016 Granada, Spain; 11 EURISTIKOS Excellence Centre for Pediatric Research, Biomedical Research Centre, University of Granada, 18071 Granada, Spain; 12 Spanish Network of Biomedical Research in Epidemiology and Public Health (CIBERESP), Granada’s Node, Institute of Health Carlos III, 28029 Madrid, Spain; 13 Granada’s Biosanitary Institute (Ibs-Granada), 18012 Granada, Spain

**Keywords:** adolescents, parents’ educational level, parents’ occupational level, birthweight, breastfeeding, adiposity, cardiorespiratory fitness, cholesterol, blood pressure, lifestyle intervention

## Abstract

We investigated which determinants (socioeconomic, early life factors, body composition changes, fitness changes and/or physical activity changes) best predicted longitudinal outcomes in cardiometabolic risk profile (Z-score change) in adolescents with OW/OB who underwent a 13-month multidisciplinary lifestyle intervention. A total of 165 adolescents (13–16 y; 46% boys) from the EVASYON study were included. Socioeconomic variables and early life factors were obtained from the medical records. Body composition was assessed using anthropometry. Fitness and physical activity were measured with field-based tests and questionnaires. Cardiometabolic risk factors (fasting glucose, HDL cholesterol, triglycerides, blood pressure and waist circumference) were derived from standard methods in the hospital. Body weight changes, sex and mother’s education were selected in the stepwise process as the most important determinants of changes in cardiometabolic risk profile (R^2^ = 0.26, *p* = 0.002; R^2^ = 0.14, *p* = 0.013; and R^2^ = 0.14, *p* = 0.017, respectively). Both boys and girls showed a lower cardiometabolic risk score with the reduction in body weight (r = 0.535, *p* = 0.009 and r = 0.506, *p* = 0.005, respectively). There was no interaction between sex and body weight change (*p* = 0.614). In conclusion, the simple measure of changes in body weight should be considered to track changes in cardiometabolic risk profile in adolescents with OW/OB.

## 1. Introduction

Overweight/obesity (OW/OB) is an important public health concern. The worldwide prevalence of OB nearly tripled between 1975 and 2016. Over 340 million children and adolescents aged 5–19 were OW or OB in 2016. Common health consequences related to OW/OB are cardiovascular diseases (the leading cause of death in 2012) and other co-morbidities, such as diabetes, musculoskeletal disorders and some types of cancer [1]. Some estimations show that more than 90 million children and adolescents might have OW or OB in 2025 [2,3]. Importantly, OW/OB is positively associated with cardiometabolic risk factors (e.g., metabolic syndrome (MetS) markers) [4,5,6,7] and a higher risk of developing cardiovascular disease later in life [8]. In this regard, socioeconomic status and several health-related indicators, such as early life factors, body composition, fitness and physical activity (PA), have been related to cardiometabolic risk profile (using MetS markers) in children and adolescents with OW/OB [9,10,11,12].

Lifestyle interventions lasting from 3 to 12 months (i.e., mainly based on nutritional education and increase in PA levels) have been proposed to improve the cardiometabolic risk profile in children and adolescents with OW/OB [13,14,15]. In this regard, the EVASYON study implemented a 13-month multidisciplinary lifestyle intervention in adolescents with OW/OB aged 13 to 16 years, recruited from five hospitals—pediatric units—in five Spanish cities [16]. Previous studies derived from the EVASYON study reported a reduction in body weight, fat mass and individual cardiometabolic risk factors (e.g., blood pressure) after 13 months of a lifestyle intervention [17,18,19]. However, to the best of our knowledge, the best predictor (e.g., socioeconomic status, early life factors, changes in body composition, fitness and PA) of longitudinal changes in the cardiometabolic risk score profile (using MetS markers), induced by a long-term multidisciplinary lifestyle intervention in adolescents with OW/OB, is poorly understood.

Therefore, in this study, we aimed to identify the socioeconomic, early life factors, body composition, fitness and PA determinants of longitudinal changes in the cardiometabolic risk score after 13 months of multidisciplinary lifestyle intervention in adolescents with OW/OB.

## 2. Materials and Methods

### 2.1. Participants and Study Design

A total of 165 adolescents (76 boys and 89 girls) were included in the data analyses. The current study falls under the umbrella of the EVASYON study, which is a multidisciplinary program implemented in adolescents with OW/OB aged 13 to 16 years, recruited from five hospitals—pediatric units—in five Spanish cities (Granada, Madrid, Pamplona, Santander and Zaragoza). The EVASYON study was conducted by different teams of multidisciplinary professionals from each hospital with small groups, from 9 to 11 adolescents, over 13 months, including twenty visits within two specific phases: (1) intensive intervention phase (9 weekly visits) and (2) extensive intervention phase (11 monthly visits). Detailed description of the EVASYON study protocol was described elsewhere [16]. Briefly, the intervention included a personalized balanced diet, a PA program and psychological support within the family. Adolescents were instructed in several motivational strategies, life and time management strategies, including PA recommendations or sleep time, nutritional advice and family involvement. All measurements were performed at baseline (0 months), at the end of the intensive intervention (2 months) and at the end of the treatment program (13 months).

Before starting the EVASYON intervention program, all candidates were screened. The inclusion criteria were: (i) 13–16 years old, (ii) OW or OB in agreement with the International Obesity Task Force age- and sex-specific body mass index (BMI, kg/m^2^) [20], (iii) Spanish or educated in Spain and (iv) no other diagnosed disease. The exclusion criterion was undergoing any pharmacological treatment.

The project followed the ethical standards recognized by the Declaration of Helsinki (reviewed in Hong Kong in September 1989 and in Edinburgh in 2000) and the EEC Good Clinical Practice recommendations (document 111/3976/88, July 1990) and current Spanish legislation regulating clinical research in humans (Royal Decree 561/1993 on clinical trials). The study was approved by the Ethics Committee of each hospital that participated in the EVASYON study and by the Ethics Committee of the Spanish Council for Scientific Research (CSIC). The study was explained to all the participants before starting, and the volunteers, parents or tutors signed an informed consent form. Their participation was completely voluntary; they did not receive any rewards.

### 2.2. Socioeconomic Variables

Parental education (father and mother) and parental occupational levels (father and mother) were extracted from the medical reports that were completed through parents’ interview. Parental education refers to the academic level indicating primary school, high school or university studies. Both parents were asked to answer a question concerning their current occupation according to the recommendations of the Spanish Society of Epidemiology [21]. Three categories of parental occupational levels were established: unskilled worker/unemployed, skilled worker and managerial, referred to as low, medium and high occupational level, respectively.

### 2.3. Early Life Factors

Birthweight (kg), size at birth (cm) and breastfeeding (none, less than 3 months, 4–6 months, 7–9 months, more than 9 months) were reported by mothers through medical interviews.

### 2.4. Body Composition Parameters

The method used for anthropometric measurements was the standardized protocol of the AVENA study [22]. The same trained researchers always obtained the measurements. Each measurement was performed three times non-consecutively, i.e., a complete set of measurements was performed and then repeated twice more. Weight and height were obtained by standardized procedures. Body mass index (BMI) was calculated as body weight (kg) divided by squared height (m^2^), classifying the adolescents as overweight or obese [23]. The classification of obesity type according to age and sex (i.e., Overweight, Obesity type I, Obesity type II and Obesity type III) was calculated with a patented Excel-based tool [24], using the cut-off points defined by Bervoets et al. [25]. Peak height velocity (PHV) was calculated from sex, age and height using Moore et al.’s equations [26].

Skinfold thicknesses were measured to the nearest 0.1 mm on the left side of the body using a skinfold caliper (Holtain Caliper; Holtain Ltd., Wales, UK) at the following sites: (1) triceps, halfway between the acromion process and the olecranon process; (2) biceps, at the same level as the triceps skinfold, directly above the center of the cubital fossa; (3) subscapular, about 20 mm below the tip of the scapula, at an angle of 45 degrees to the lateral side of the body; (4) suprailiac, about 20 mm above the iliac crest and 20 mm toward the medial line; (5) thigh, in the midline of the anterior aspect of the thigh, midway between the inguinal crease and the proximal border of the patella; (6) calf, at the level of maximum calf circumference, on the medial aspect of the calf. The sum of 4 skinfolds included the biceps, triceps, subscapular and suprailiac, and the sum of 6 skinfolds included all the skinfolds measured [22].

Fat mass (FM) in kg and fat free mass (FFM) in kg were calculated from the adolescent’s total body weight and the percentage of total body fat that was obtained using the equations reported by Slaughter et al. for children and adolescents [27].

### 2.5. Fitness and Physical Activity

#### 2.5.1. Cardiorespiratory Fitness

Cardiorespiratory fitness (CRF) was assessed using the progressive 20 m shuttle run test published by Léger and Lambert in 1982 and revised in 1988 [28], where participants run as long as possible back and forth across a 20 m space at a specified music protocol that becomes 0.5 km/h faster each minute or stage. The last 0.5 stage completed is the individual score, and VO_2_max was estimated with the Léger equation (i.e., CRF relative to body weight). In addition, CRF relative to FFM was calculated from individual absolute oxygen consumption divided by FFM.

#### 2.5.2. Muscular Strength

A handgrip strength test was performed with the adolescents in a standard bipedal position and with the arm in complete extension without touching any part of the body with the hand dynamometer (TKK 5101; Takei, Tokyo, Japan). The dynamometer was adjusted for sex and hand size for each adolescent. The test was performed twice using both hands alternatively, allowing short rest between the measures. The final score is the average of the two hands, taking the maximum score from each attempt [29]. The handgrip strength test provides information about the maximal isometric strength that can be generated mainly by the hand and the arm. Relative handgrip strength (kg/kg) was calculated by dividing the original handgrip strength by the adolescents’ total body weight. Standing long jump (cm) was performed in the standing position; the adolescent jumps as far as possible, trying to land with both feet together. The score is the maximum distance between the last heel mark and the take-off line of two attempts. The standing broad jump assesses lower-limb explosive strength.

#### 2.5.3. Physical Activity

PA levels were obtained from the Physical Activity Questionnaire for Adolescents (PAQ-A) [30]. The adolescents were asked about their frequency of PA during the week and weekend days and to compute the score ranging from 1 to 5 points, 1 being the lowest level and 5 the highest level of PA.

### 2.6. Cardiometabolic Risk Factors

#### 2.6.1. Fasting Glucose, Triglycerides and High-Density Lipoprotein

Fasting blood samples were taken in the morning (after a 10 h overnight fast); the adolescents went to the hospital for blood sampling between at 8 and 10 a.m. Blood samples were collected by puncture of the cubital vein (21.5 mL). For biochemical analyses, serum was separated from plasma by centrifugation, divided into aliquots and frozen and stored at −80 °C until analysis. Fasting glucose, triglycerides and high-density lipoproteins cholesterol (HDLc) were analyzed by the biochemical auto analyzer Olympus model AU2700 (Olympus, Melville, NY, USA). All serum analyses were performed at the end of the study in order to have the three samples from each subject analyzed in the same run to avoid systematic errors. The coefficients of variance of the lipid variables were 3% for triglycerides and 2% for HDLc [31].

#### 2.6.2. Blood Pressure

Resting blood pressure was obtained using the left arm and measured using a validated digital automatic blood pressure monitor (OMRON M6, OMRON HEALTH CARE Co., Ltd., Kyoto, Japan) according to the International Protocol of the European Society of Hypertension [32]. For this study, the mean arterial blood pressure (MAP) in mmHg was used: MAP = Diastolic Blood Pressure + [0.333 × (Systolic Blood Pressure − Diastolic Blood Pressure)], according to the National High Blood Pressure Education Program Working Group on High Blood Pressure in Children and Adolescents [33].

#### 2.6.3. Waist Circumference

The waist circumference (WC) was measured with an inelastic tape to the nearest millimeter, with the subject upright; the measuring tape was applied horizontally midway between the lowest rib margin and the iliac crest, at the end of a gentle exhalation [22].

#### 2.6.4. Cardiometabolic Risk Score Using MetS

The cardiometabolic risk Z-score was calculated using the standardized Z-scores for each cardiometabolic risk factor at different time points, as follows: (Z-score fasting glucose + Z-score triglycerides + Z-score reverted HDLc + Z-score MAP + Z-score WC)/5. Higher scores indicate greater cardiometabolic risk [34].

### 2.7. Statistical Analysis

The participants’ descriptive data are presented as means ± standard deviation or frequency and percentages for continuous and categorical variables, respectively. Student’s *t*-tests and chi-squared tests were performed to investigate baseline differences for continuous and categorical variables, respectively, between boys and girls. Stepwise linear regression was performed to test the strongest potential determinants (i.e., socioeconomic, early life factors, body composition changes, fitness changes and PA changes) of the changes in the cardiometabolic risk score (Z-score change). The first model included changes in the above-mentioned variables in the intensive intervention phase. In contrast, the second model included changes in variables in the extensive intervention phase. All analyses were conducted using the Statistical Package for Social Sciences (SPSS, v. 21.0, IBM SPSS Statistics, IBM Corporation, Armonk, NY, USA). The significance level was set at 0.05. Figures were depicted using the GraphPad Prism software for Windows (version 5.0.0, GraphPad Software, San Diego, CA, USA).

## 3. Results

The descriptive characteristics of the participants at baseline are shown in Table 1. The study sample presented a chronological age of 14.48 ± 1.23 years and a biological maturation age (PHV) of 1.57 ± 1.21 years, with 54% girls. The adolescents’ prevalence according to parental education was 37% for primary school for both parents, 37% for high school for fathers and 39% for mothers, and for university studies, 26% and 24% for fathers and mothers, respectively. With respect to the occupational level, fathers and mothers showed higher percentage in the lower professional level (>40%) than in the medium (>35%) or higher level (>16%). At baseline, obesity type I was the prevalent degree of obesity (44%) in the study sample, followed by overweight and obesity type II (25% and 21%, respectively), and finally, obesity type III (10%). Boys presented higher cardiorespiratory and muscular fitness, body weight, birth weight and adiposity than girls (*p* < 0.05). Girls reported higher PHV and fat free mass percentage than boys (*p* < 0.05).

Table 2 presents stepwise linear regressions between the potential determinants (i.e., socioeconomic, early life factors, body composition changes and fitness and PA changes) and the changes in the cardiometabolic risk score (Z-score change) in the intensive and the extensive intervention phases. None of the potential determinants was selected in the stepwise model in the intensive intervention phase. Meanwhile, body weight changes (post–pre intervention values), sex and mother’s education were entered in the stepwise model as significant determinants in the extensive intervention phase (R^2^ = 0.26, *p* = 0.002; R^2^ = 0.14, *p* = 0.013; and R^2^ = 0.14, *p* = 0.017, respectively). Body weight changes explained 26% of the variations in the cardiometabolic risk score (Z-score change), while the inclusion of sex and mother’s education in the model showed an additional 28% (14% for sex and 14% for mother’s education, respectively) of the explained variations in the cardiometabolic risk score (Z-score change).

**Figure 1:**
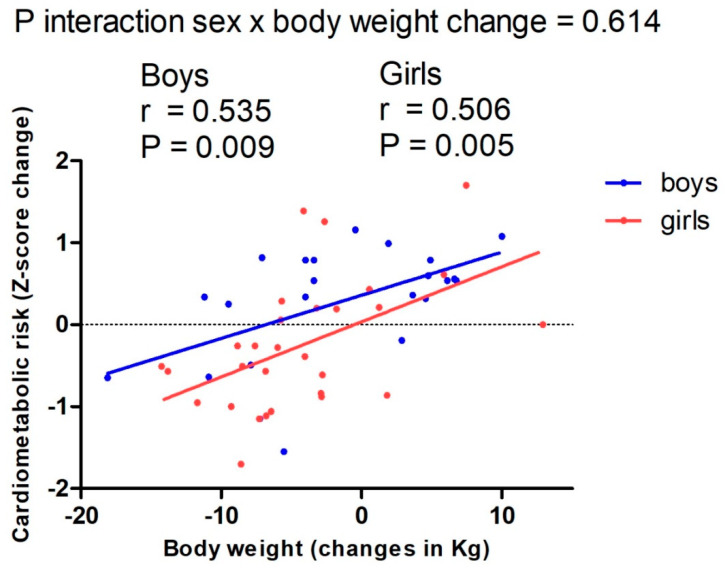
Scatter plot showing the association between changes in body weight and the cardiometabolic risk score (Z-score change) stratified by sex at the end of the extensive intervention phase.

Figure 1 presents the association according to sex between changes in body weight and changes in the cardiometabolic risk score (Z-score change) at the end of the extensive intervention phase. Both boys and girls showed a lower cardiometabolic risk score with the reduction in body weight (r = 0.535, *p* = 0.009 and r = 0.506, *p* = 0.005, respectively). There was no interaction between sex and body weight change (*p* = 0.614). Girls presented lower cardiometabolic risk scores at any level of body weight change compared to boys.

## 4. Discussion

The EVASYON study is a multi-center project that aims to investigate the effect of a 13-month multidisciplinary lifestyle intervention on different health-related outcomes (e.g., body composition, cardiometabolic risk factors, among others) in adolescents with OW/OB aged 13 to 16 years [16]. In this regard, different studies from the EVASYON project reported an improvement in body weight, fat mass and cardiometabolic risk factors after 13 months of a lifestyle intervention in adolescents with OW/OB [17,18,19]. However, the determination of the best predictor from a broad set of health-related outcomes (socioeconomic, early life factors, body composition changes, fitness changes and PA changes) of longitudinal changes in the cardiometabolic risk profile (Z-score change) in adolescents with OW/OB remains, to the best of the authors’ knowledge, unexplored. In this regard, the main findings of the present study were: (1) none of the potential determinants (i.e., socioeconomic, early life factors, body composition changes, fitness changes and PA changes) of the changes in the cardiometabolic risk score (Z-score change) were relevant in the intensive phase of the intervention; (2) instead, body weight changes, sex and mother’s education predicted longitudinal changes in the cardiometabolic risk score (Z-score change) in adolescents with OW/OB in the extensive phase of the intervention. Reductions in body weight were associated with a lower cardiometabolic risk in boys and girls. Moreover, for the same reductions in body weight, the cardiometabolic risk was lower in girls compared with boys.

Low socioeconomic status is associated with a higher cardiometabolic risk and development of OW/OB during the early stages of life (i.e., from 0 to 15 years old) [35], and it seems to predict a higher risk of MetS in adulthood [36]. Although the associations between socioeconomic status and cardiometabolic risk are clearly detected in adults [37], the study results in adolescents are contradictory. Puolakka et al. [36] found that family socioeconomic status was associated with MetS and, in line with our results, Loucks et al. [38] found no relationship. Different methodological aspects between studies could partially explain these discrepancies (e.g., demographic heterogeneity in the study populations and sample size, SES measured by annual income [36] or the family income plus years of parental education [38]).

Additionally, parental education and occupation (low education level and unemployment, respectively) are related to the development of OW/OB and an adverse cardiometabolic risk profile in the pediatric population [9,35]. In this regard, Iguacel et al. [9] reported that mother’s education was strongly associated with cardiometabolic risk in adolescents, independently of lifestyle. Additionally, the mother’s education may impact children’s adiposity due to the mother’s influence on the diet during the early stage of life in kids and the duration of breastfeeding [39,40]. Similarly, we observed that mother’s higher education was inversely associated with cardiometabolic risk (i.e., a higher level of education was related to a lower cardiometabolic risk) in adolescents with OW/OB. However, in our study, reductions in body weight were a stronger determinant of longitudinal changes in cardiometabolic risk than socioeconomic status variables.

Early life factors are also associated with adiposity and cardiometabolic health in adulthood [41,42,43,44,45,46,47]. Higher birth weight was associated with early development of insulin resistance and MetS [44] and increased risk of obesity [45]. Additionally, a lower birth weight was related to later risk of cardiovascular disease, obesity and diabetes [43]. In conclusion, low and high birth weights impact subsequent cardiometabolic health [41]. For size at birth, especially lower size was associated with subsequent cardiometabolic health [42]. In our study, weight and size at birth were not associated with cardiometabolic risk factors. Finally, there is also a relationship between breastfeeding and later diseases; a short period or absence of breastfeeding seems to be a risk factor for MetS [43,46]. Conversely, and along the same lines as Yakubov et al. [47], we found no association between cardiometabolic risk and any of the breastfeeding periods.

Interestingly, body weight reduction rather than changes in body composition indices (e.g., adiposity), fitness or PA was the strongest predictor of changes in the cardiometabolic risk profile (Z-score change) in adolescents with OW/OB. Of note, Ortega et al. reported that body mass index was a better predictor of cardiovascular disease mortality than body composition indices measured using hydrostatic weighing in a cohort of 60.335 participants followed up for a mean follow-up period of 15.2 years [48]. Similarly, we observed that the inexpensive and simple measure of body weight reflects the changes in the cardiometabolic risk profile (Z-score change) induced by a 13-month multidisciplinary lifestyle intervention better than any body composition indices. However, contrary to Ortega et al. [48], we did not use gold standard methods to determine body composition indices. Importantly, we believe that our findings are interesting for implementing a 13-month multidisciplinary lifestyle intervention in adolescents with OW/OB. A 30-year cohort study showed that the negative impact of higher body weight on cardiometabolic risk originates early in life [49].

Fitness and PA are inversely associated with cardiometabolic risk in youth [50,51]. Likewise, fitness may attenuate the adverse association between high body weight/adiposity and cardiometabolic risk in children and adolescents [34,52]. However, in the current study, changes in fitness and PA were not predictors of changes in cardiometabolic risk (Z-score change) in adolescents with OW/OB. In this regard, gold standard methods, such as indirect calorimetry and accelerometer, were not used in our study to assess fitness and PA, respectively. On the other hand, sex was the second determinant (the first determinant in the stepwise regression model was the reduction in body weight) of the changes in the cardiometabolic risk profile (Z-score change) in adolescents with OW/OB. We did not detect an interaction effect between changes in body weight and sex. Otherwise, for the same reductions in body weight, girls showed a lower cardiometabolic risk profile (Z-score) than boys. Interestingly, Barstad et al. examined sex differences in cardiometabolic risk factors in adolescents with obesity [53]. Specifically, girls presented lower levels of TG, blood pressure and higher HDL than boys with obesity, although PA levels were similar [48,53].

Our study presents some limitations that should be acknowledged. First, body composition indices were determined using skinfolds, and other methods, such as dual energy X-ray absorptiometry and air displacement plethysmography, may be more accurate. Second, PA and fitness were not measured using gold standard methods, such as accelerometry and indirect calorimetry. Third, PA questionnaires had to be applied in different seasons, and PA behavior could be affected by the seasonal effect. Fourth, our study lacks a control group to assess longitudinal changes induced by the lifestyle intervention in adolescents with OW/OB. Despite these limitations, our study tested several potential determinants (socioeconomic status, early life factors, body composition changes, fitness changes and PA changes) of changes in the cardiometabolic risk profile (Z-score) after a 13-month multidisciplinary lifestyle intervention in a cohort of 165 adolescents with OW/OB.

## 5. Conclusions

In the context of a 13-month multidisciplinary lifestyle intervention, changes in body weight rather than socioeconomic status, early life factors, body composition changes, fitness changes and PA changes were better predictors of changes in the cardiometabolic risk profile (Z-score change) in adolescents with OW/OB. In addition, the cardiometabolic risk profile was more favorable in girls than in boys for the identical decrease in body weight. In summary, the simple and inexpensive measure of body weight should be considered to track changes in the cardiometabolic risk profile in adolescents with OW/OB.

## Figures and Tables

**Table 1:** Descriptive characteristics of the participants at baseline.

Variables	All Samples			Boys			Girls			*p*-Value
**Socioeconomic Variables**										
Sex [n (%)]	165	(100%)		76	(46%)		89	(54%)		NA
Age (years)	14.48	±	1.23	14.40	±	1.19	14.55	±	1.25	0.42
PHV (years)	1.57	±	1.21	0.79	±	0.99	2.22	±	0.97	**<0.01**
Father’s education										0.64
Primary School [n (%)]	56	(37%)		27	(38%)		29	(36%)		
High School [n (%)]	55	(37%)		23	(33%)		32	(40%)		
University [n (%)]	39	(26%)		20	(28%)		19	(24%)		
Mother’s education										0.93
Primary School [n (%)]	54	(37%)		25	(35%)		29	(38%)		
High School [n (%)]	57	(39%)		18	(39%)		29	(38%)		
University [n (%)]	36	(24%)		28	(25%)		18	(24%)		
Father’s occupational level										0.55
Low [n (%)]	61	(41%)		27	(40%)		34	(42%)		
Medium [n (%)]	51	(35%)		21	(31%)		30	(37%)		
High [n (%)]	36	(24%)		19	(28%)		17	(21%)		
Mother’s occupational level										0.72
Low [n (%)]	76	(49%)		33	(46%)		43	(52%)		
Medium [n (%)]	54	(35%)		26	(36%)		28	(34%)		
High [n (%)]	25	(16%)		13	(18%)		12	(14%)		
**Early life factors**										
Birth weight (kg)	3.35	±	0.54	3.49	±	0.61	3.22	±	0.45	**<0.01**
Size at birth (cm)	50.65	±	2.65	50.78	±	2.57	50.52	±	2.74	0.57
Breastfeeding										0.71
None [n (%)]	20	(14%)		12	(17%)		8	(10%)		
<3 months [n (%)]	69	(47%)		31	(46%)		38	(48%)		
4–6 months [n (%)]	36	(24%)		15	(22%)		21	(26%)		
7–9 months [n (%)]	12	(8%)		6	(9%)		6	(8%)		
>9 months [n (%)]	10	(7%)		4	(6%)		6	(8%)		
**Body composition parameters**										
Weight (kg)	86.23	±	17.12	90.36	±	17.16	82.77	±	16.40	**<0.01**
Height (cm)	164.38	±	7.95	167.90	±	7.87	161.44	±	6.78	**<0.01**
BMI (kg/m^2^)	31.76	±	5.07	31.87	±	4.73	31.66	±	5.37	0.80
Obesity types										0.43
Overweight [n (%)]	39	(25%)		14	(20%)		25	(30%)		
Obesity type I [n (%)]	68	(44%)		33	(47%)		35	(41%)		
Obesity type II [n (%)]	33	(21%)		18	(25%)		15	(18%)		
Obesity type III [n (%)]	15	(10%)		6	(8%)		9	(11%)		
Sum of 4 skinfolds (mm)	112.11	±	19.89	112.28	±	18.68	111.97	±	20.96	0.92
Sum of 6 skinfolds (mm)	186.44	±	25.30	186.21	±	24.54	186.63	±	26.05	0.91
Fat mass (kg)	28.24	±	7.97	33.36	±	8.28	23.92	±	4.32	**<0.01**
Fat mass (%)	32.57	±	5.19	36.62	±	3.94	29.15	±	3.32	**<0.01**
Fat free mass (kg)	58.05	±	12.03	57.01	±	10.01	58.93	±	13.50	0.32
Fat free mass (%)	67.43	±	5.18	63.38	±	3.94	70.84	±	3.31	**<0.01**
**Fitness and physical activity**										
CRF (Stages)	2.90	±	1.45	3.36	±	1.54	2.49	±	1.24	**<0.01**
CRF relative to BW (ml/kg/min)	35.61	±	4.17	36.95	±	4.02	34.40	±	3.95	**<0.01**
CRF relative to FFM (ml/kg/min)	53.18	±	7.85	58.27	±	5.62	48.62	±	6.68	**<0.01**
Handgrip strength (kg)	28.82	±	7.46	32.09	±	8.40	26.00	±	5.11	**<0.01**
Relative handgrip strength (kg/kg)	0.34	±	0.07	0.36	±	0.07	0.32	±	0.07	**<0.01**
Standing long jump (cm)	124.31	±	25.16	132.89	±	25.25	117.01	±	22.80	**<0.01**
Physical Activity levels (PAQ-A)	1.62	±	1.06	1.72	±	1.16	1.53	±	0.97	0.28
**Cardiometabolic risk factors**										
Fasting Glucose (mg/dL)	84.15	±	8.37	83.97	±	8.20	84.30	±	8.57	0.81
Triglycerides (mg/dL)	89.01	±	41.74	88.27	±	39.06	89.74	±	44.55	0.84
HDL cholesterol (mg/dL)	46.04	±	10.77	44.86	±	10.20	47.22	±	11.27	0.21
Mean arterial blood pressure (mmHg)	85.01	±	10.33	85.45	±	9.42	84.62	±	11.12	0.65
Waist circumference (cm)	98.71	±	12.79	104.00	±	11.02	94.25	±	12.53	**0.01**

Table 1 legend: NA, not applicable; PHV, peak height velocity; BMI, body mass index; Sum of 4 skinfolds (Biceps, Triceps, Subscapular, Suprailiac); Sum of 6 skinfolds (Biceps, Triceps, Subscapular, Suprailiac, Thigh, Calf); CRF, cardiorespiratory fitness; BW, body weight; FFM, fat free mass; PAQ-A, physical activity questionnaire for adolescents; HDL, high-density lipoprotein. *p*-values show differences between boys and girls. Bold numbers indicate *p*-value < 0.05.

**Table 2:** Stepwise linear regressions of potential determinants of longitudinal changes in the cardiometabolic risk score (Z-score change) in adolescents with overweight/obesity.

Intervention Phase	Significant Determinants (*p* < 0.05)	β	R2 of Change	*p*-Value
Intensive phase	Irrelevant determinants were included	NA	NA	NA
Extensive phase	Weight (Δ)	0.534	0.261	0.002
	Sex (1 = boys; 2 = girls)	−0.409	0.144	0.013
	Mother’s education (1 = Primary School; 2 = High School; 3 = University)	−0.388	0.141	0.017

Table 2 legend: The potential determinants of longitudinal changes in the cardiometabolic risk, presented in Table 2, were included as predictors in the stepwise models. The cardiometabolic risk score (fasting glucose, triglycerides, HDL cholesterol, blood pressure and waist circumference) differences (i.e., Δ changes; post–pre) were included as the dependent variables.

## Data Availability

The data presented in this study are available on request from the corresponding author. The data are not publicly available due to restrictions concerning privacy and ethical reasons.
